# High dielectric constant and high breakdown strength polyimide *via* tin complexation of the polyamide acid precursor†

**DOI:** 10.1039/d1ra06302b

**Published:** 2022-03-23

**Authors:** Abdullah Alamri, Chao Wu, Shamima Nasreen, Huan Tran, Omer Yassin, Ryan Gentile, Deepak Kamal, Rampi Ramprasad, Yang Cao, Gregory Sotzing

**Affiliations:** Institute of Materials Science, University of Connecticut USA yang.cao@uconn.edu g.sotzing@uconn.edu; Electrical Insulation Research Center, Institute of Materials Science, University of Connecticut USA; School of Materials Science and Engineering, Georgia Institute of Technology USA; Department of Chemistry, University of Connecticut USA

## Abstract

Polymer dielectrics with ultra-high charge–discharge rates are significant for advanced electrical and electronic systems. Despite the fact that polymers possess high breakdown strength, the low dielectric constant (*k*) of polymers gives rise to low energy densities. Incorporating metal into polyimides (PI) at the polyamic acid (PAA) precursor stage of the synthetic process is a cheap and versatile way to improve the dielectric constant of the hybrid system while maintaining a high breakdown strength. Here, we explore inclusion of different percentages of Sn as a coordinated complex in a polyimide matrix to achieve metal homogeneity within the dielectric film to boost dielectric constant. Sn–O bonds with high atomic polarizability are intended to enhance the ionic polarization without sacrificing bandgap, a measurable property of the material to assess intrinsic breakdown strength. Enhancements of *k* from *ca.* 3.7 to 5.7 were achieved in going from the pure PI film to films containing 10 mol% tin.

## Abstract 2

Polyimide with high dielectric constant and breakdown strength is synthesized via tin complexation of the polyamide acid precursor. Sn–O bonds with high atomic polarizability are intended to enhance the ionic polarization without sacrificing bandgap.

## Graphical abstract

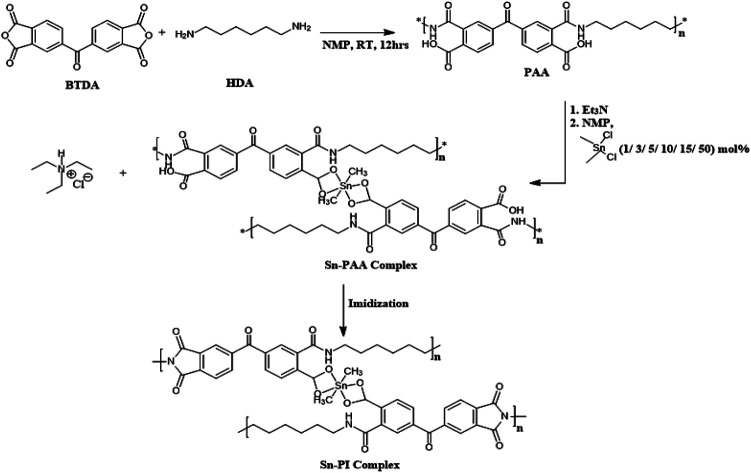

## Introduction

The effective dielectric medium for energy conversion and storage has become increasingly important for electrical and electronic systems.^1–5^ Capacitive energy storage with an ultra-high charging-discharging rate is essential for many applications, such as pulsed power sources, high-frequency inverters, and medical defibrillators. The simultaneous demand to improve the payload efficiency and miniaturize the volume of the energy storage system call for capacitors with high energy densities. The energy density *U*_e,_ is quadratically correlated to the maximum operational electric field strength *E*, and proportional to the dielectric constant *ε*_r_, times the vacuum permittivity *ε*_o_, for linear dielectric materials, all in accordance with the equation, *U*_e_ = (*ε*_r_*ε*_o_*E*^2^)/2.^6–10^

Organic polymer dielectrics have improved the processing scalability, lowered the weight, and enhanced the graceful failure mode of capacitors. More importantly, organic polymers possess a high breakdown strength, which is the foremost significant parameter for higher energy density. However, there is always a limitation of using organic polymers for capacitive energy storage due to their low dielectric constants. The state-of-the-art commercially available polymer film, biaxially oriented polypropylene (BOPP), is used for capacitors due to its ultra-low dielectric loss (∼0.0002) and conduction loss.^11,12^ The moderate energy density of ∼5 J cc^−1^ for BOPP at a dielectric breakdown field strength of ∼700 MV m^−1^ arising from the low dielectric constant (∼2.2) limits its further development for electronic and pulsed power applications, such as electric vehicles and devices requiring high payload efficiencies, respectively.^13,14^ One of the challenges to improving the dielectric constant of an insulator arises from maintaining a low dielectric loss for high charge–discharge efficiency, while simultaneously maintaining a large bandgap to ensure high breakdown strength. This proves problematic, as one of the significant sources of the total dielectric constant is the electronic portion, which is inversely correlated to the bandgap, of which essentially controls the intrinsic breakdown strength of dielectric materials.^5^

Searching for a new route of developing dielectric materials is crucial and challenging, in that it must meet the optimum electrical properties without largely sacrificing one over another.^15^ Steps have been taken to improve the dielectric constant by introducing nano-fillers like metal oxides which rely on ionic polarization.^12,16–20^ The accompanying agglomeration of inorganic fillers leads to distortion of the electric field and lowers the breakdown strength. Metals were also incorporated into polymers in different ways to improve the dielectric constant for stronger polarizability, in the form of Sn, Cd, or Zn containing polyesters.^15,21,22^ An effective way to uniformly disperse metals and eliminate the incompatibility of metal-polymer interfaces was to chemically bond the metal into the network of polymer chains. Polymers containing metal atoms in the backbone have been newly developed as dielectric materials.^5,15,21,22^ After thorough structural and electrical experimentation and design, it has been found that the newly developed organometallic tin polyester systems exhibit high dielectric constants (6.1–6.8) and have excellent agreement with their computational predictions.^15,21,22^ However, similar to BOPP, the thermal stability of Sn-polyesters still needs to be further improved as the ultra-fast charging-discharging process can produce a large amount of heat and increase the temperature of the energy storage device.^21,23^

**Scheme 1:**
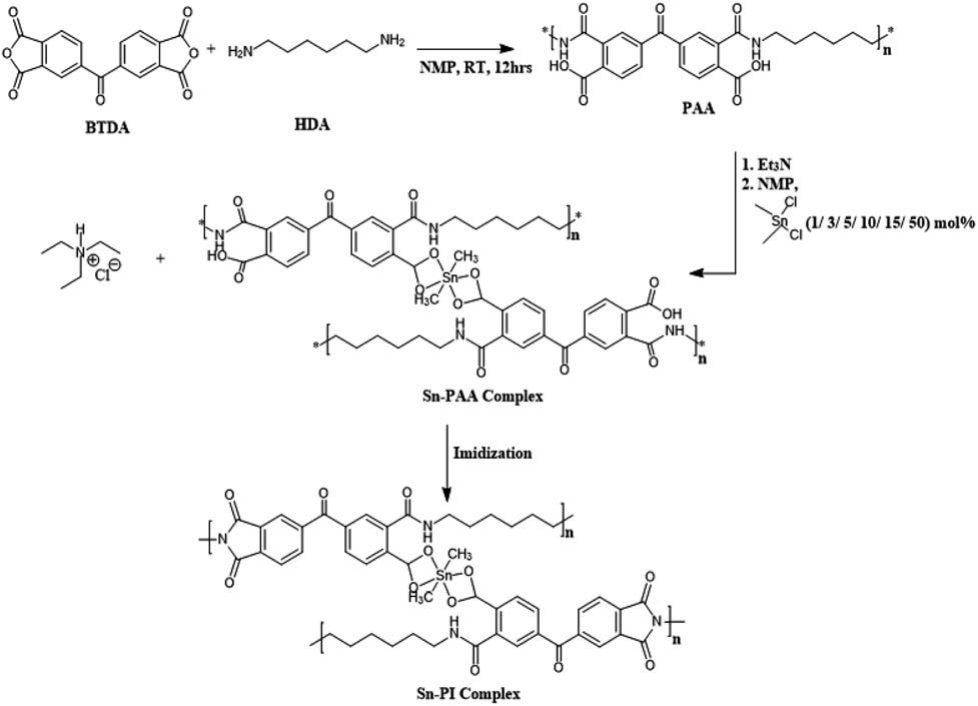
Synthetic route of Sn-PAA complex, showing cross-linked DMT polyimide motifs.

PI's are historically well-known for their high temperature resistance, chemical stability, and excellent mechanical and electrical performances.^24–26^ They also have shown excellent film formation ability with moderate dielectric constants, typically around 3. Here, we explored a new route of incorporating Sn into the organic polymers with the goals of improving the dielectric constant, breakdown strength, and energy density of the organometallic system *via* chemically bonding Sn into the PI backbone. Our strategy was to utilize the Sn–O bond to enhance the ionic polarization. It can also improve the film morphology by disrupting the polymer chain packing. Systematic characterizations were then performed to reveal the role of Sn in polarization, electrical breakdown, and film processing of the polyimide-metal polyester hybrid systems. We synthesized the PAA's first and then produced coordination complexes to crosslink the PAA's with different percentages of Sn metal inclusion forming the resultant PAA Sn-polyesters. The remaining PAA functional groups without coordinated Sn were imidized, and all were film cast to produce the PI/PAA Sn polyester blend systems. Scheme 1 below illustrates the synthetic route and reaction conditions. Different mole % ratios (1%, 3%, 5%, 10%, 15%, and 50%) of dimethyltin dichloride (DMT) based on the moles of PAA were used to form different amounts of Sn metal coordinated PAA complex. The polymers are named (Sn1-PAA, Sn3-PAA, Sn5-PAA, Sn10-PAA, Sn15-PAA, and Sn50-PAA, respectively, where numbers indicate the mol% ratios of DMT with respect to PAA.

## Results and discussion

### Structural characterization

**Fig. 1:**
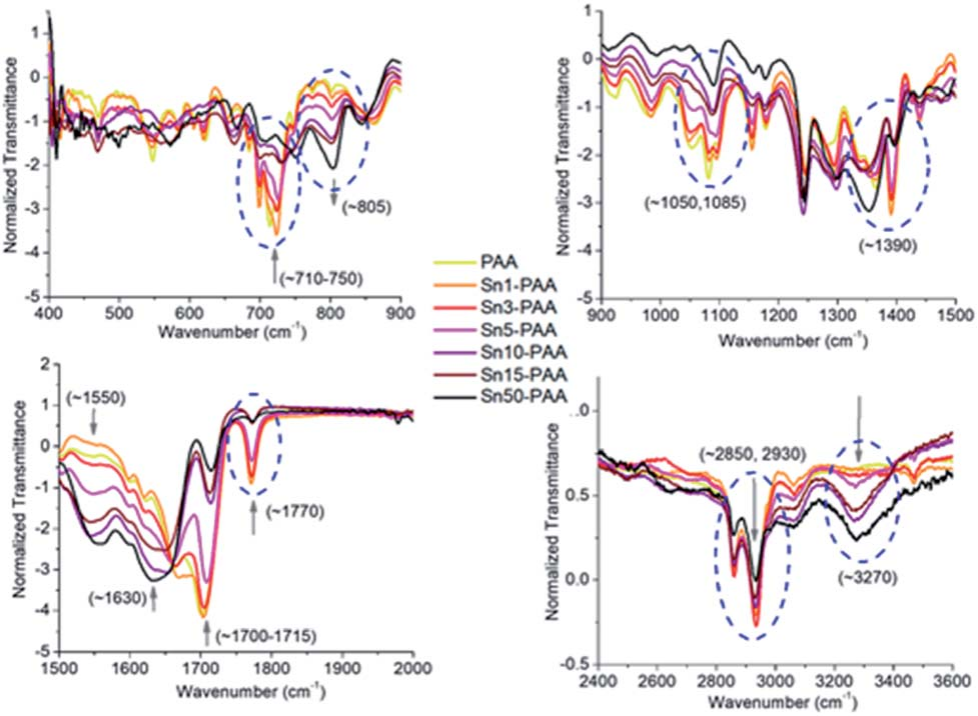
Overlay of the same set of FTIR spectra segmenting different important frequency regions.

The structure of PAA and PAA coordinating Sn metal was characterized by Fourier transform infrared spectroscopy (FTIR). Fig. 1 shows the FTIR spectra of PAA coordinating different percentages of Sn metal. The imide (–C=O) bands at 1770 cm^−1^ (asymmetric) and 1708 cm^−1^ (symmetric) are decreased, while the amide (–C=O) band at ∼1630 cm^−1^ is increased and broadened with the increased amounts of Sn.^27^ At the same time the ∼1390 cm^−1^ peak for the C–N stretch band is decreased by the addition of Sn. Typical absorption bands for aromatic imides can be observed at 1390, 1710, and 1770 cm^−1^ in all spectra, respectively, shown with their decreasing transmittance by the addition of increasing Sn inclusion. The gradual decrease of these characteristic imide band peaks inversely correlates with an increase in the Sn concentration of the polymer samples, indicating the lowering presence of the imide linkage and increasing presence of the amide moiety of the newly formed Sn PAA esters.

### Theoretical aspects of model compound

Density Functional Theory (DFT) is a powerful tool to study a wide range of material properties beginning at the atomic level. Among the indicators of the formation of possible Sn coordination complex structures, thermodynamic stability is perhaps one of the properties that can be reliably accessed using this computational method. In this work, we used the Vienna *ab initio* Software Package (VASP)^28^ for our DFT calculations. We found four possible Sn coordination complex structures, shown in Fig. 1S.† Among them, structure C is thermodynamically most stable, which is at about 0.31 eV below the next arrangement, *i.e.*, structure B. Due to their proximity, it can be argued that both structures can prevail at room temperature, at which *k*_b_*T* is just about 0.025 eV per atom, being much smaller than the energy difference between structures B and C.

### Morphological features

The samples were investigated for their morphological features by SEM shown in Fig. 2S in the ESI.† Elemental mapping of different films showed the homogeneous distribution of Sn throughout the matrix. The green dots represent the oxygen distribution, whereas the red dots represent the Sn. The ratio of red dots representing the oxygen increases with increasing amounts of Sn content; on the contrary, there were no significant changes in the distribution of the red dots. The reason is that the amount of oxygen in the matrix was the same whether it forms the complex with Sn or not. No microphase separation is observed in any of the compositions, indicating the nano-level homogeneous distribution of the DMT particles.

Whether the Sn forms any oxide as dimethyl tin oxide (DMTO) inside the polymer or remains as a complex can be demonstrated in comparison with the actual mixture of DMTO and PAA. Physical mixtures of PAA and commercial DMTO were prepared with different proportions, namely M1, M3, M5, M15, and M50, where numbers indicate the percentage weight ratios of DMTO with respect to PAA. The XRD spectra are shown in Fig. 3S,† which suggest that the mixtures and Sn coordinating polymers had different structural and morphological features. All polymer films with Sn coordination complexes exhibited amorphous characteristics, even upon the addition of a 1 : 1 weight ratio (Sn50-PAA) of Sn to PAA. There is a change in the amorphous peak going from solely PAA to Sn50-PAA. The broad amorphous peaks of the polymer samples shifted to lower angles gradually by the addition of Sn. This peak can be related to the presence of Sn.^29^ By the addition of DMTO, the PAA films (M series) gradually changed to crystalline which is indicated by the sharp characteristic peaks seen by XRD. A 1 : 1 ratio of DMTO to PAA (M50) closely resembles the pure DMTO, which indicates that the amorphous nature of the PAA film is completely suppressed by the addition of DMTO and becomes more crystalline. This is opposite to what we observed in the polymer films with Sn coordination complexes present. Therefore, we have found the internal structure of the films cast as polymer *vs.* mixture to be different from one another.

### Thermal characterization

Thermal stability for all polymer samples was characterized by Thermal Gravimetric Analysis (TGA) under nitrogen atmosphere. The TGA curves for all samples are shown in Fig. 4S.† The 20% weight loss temperature (*T*_d20%_) is around 440 °C for PAA and drops as a function of increasing metal content. The lowest degradation temperature at 20% weight loss was found for the system with 1 : 1 ratios of Sn to PAA, for which we assumed all the Sn coordinated with the COO- groups. DSC thermograms of the samples were shown in Fig. 5S,† where it can be found that the incorporation of Sn increases the *T*_g_ of the samples. Up to a threshold concentration (10%), the Sn is acting as a PI chain disrupter by making coordination organometallic complexes within the linked sites and causing the distribution of the Sn to be more homogenous. On the other hand, the increased metallic entity in the polymer bulk increases the crystallinity of the polymer by forming a three-dimensional network within the system. The 1 : 1 ratio of Sn to PAA where the content of Sn is 50% with respect to PAA to fully coordinate with PAA showed no *T*_g_ values up to 200 °C. In this system all Sn is coordinated with the PAA, making it more crystalline and brittle in nature rather than being capable of existing as a free-standing film. Table 1S (ESI)† shows the *T*_g_ values and the *T*_d20%_ values for PAA and PAA with different amounts of Sn metal incorporation.

### Dielectric constants

**Fig. 2:**
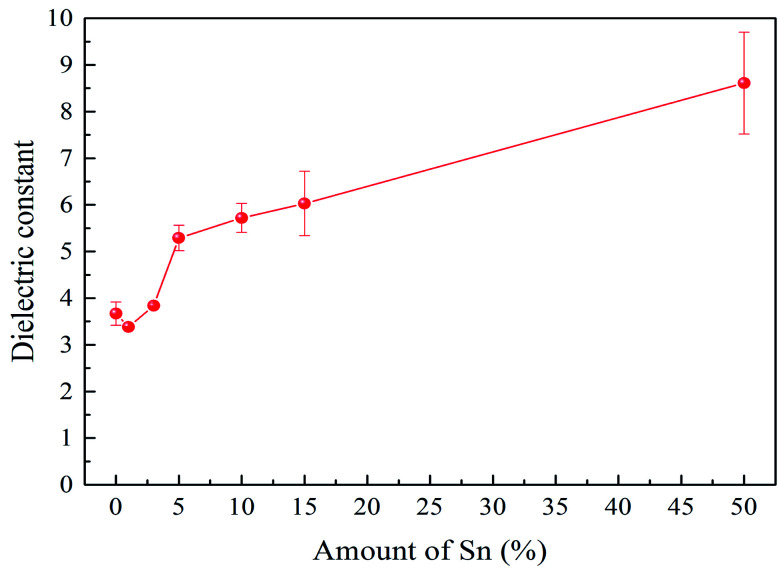
Plot of dielectric constants measured at RT and 1 kHz with different mol% of Sn metal inside the polymer.

**Fig. 3:**
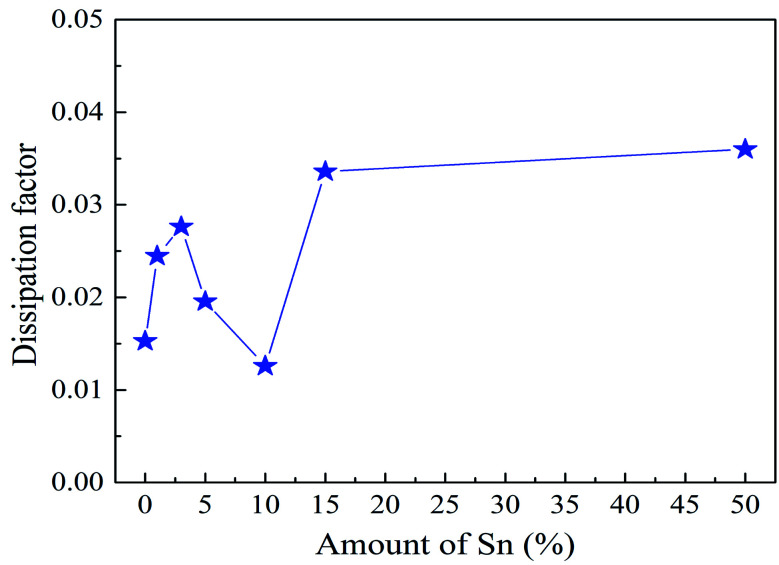
Dissipation factor with different mol% of Sn inside the polymer measured at RT and 1 kHz.

Dielectric constants and dissipation factors have been investigated through frequency domain spectroscopy at room temperature from 20 Hz to 1 MHz. As dielectric constants had no significant change in the frequency range investigated, we chose the dielectric constants at 1 kHz as a typical value to concisely show the variation trend of dielectric constants with the Sn amount varying. Dielectric constants at 1 kHz as a function of the amount of Sn are illustrated in Fig. 2. The error bars denote the standard deviation of dielectric constants measured on different samples. A slight decrease was found from PAA without Sn to PAA with 1% Sn. Although the stronger polarizability of Sn is beneficial to increase the dielectric constant, the effect of crosslinking possibly dominated when the mol% of Sn increased from 0 to 1%. The increased crosslinking density can also restrict the movement of polymer chains, contributing to a lower dielectric constant. Further increasing the mole percentage of Sn to 5% significantly increased the dielectric constants from 3.4 ± 0.1 to 5.3 ± 0.3, indicating that the enhanced ionic polarization originating from the incorporation of Sn governed the change in dielectric constants. Subsequently, the dielectric constant continued to gradually increase with increasing Sn content. The uniform distribution of Sn *via* chemically bonding it in the polymer structure significantly improved the dielectric constants, while maintaining a low polarization loss. The dissipation factors representing the dielectric loss are shown in Fig. 3. The decrease of the dissipation factor with the Sn amount from 3% to 10% is probably due to the crosslinking effect of Sn. The increased crosslinking density restricted the polymer chain movement and thus decreased the dielectric loss (dissipation factor). Further increasing of the Sn amount made the film brittle, contributing to the subsequent increase of the dissipation factor.

### DC breakdown strength

**Fig. 4:**
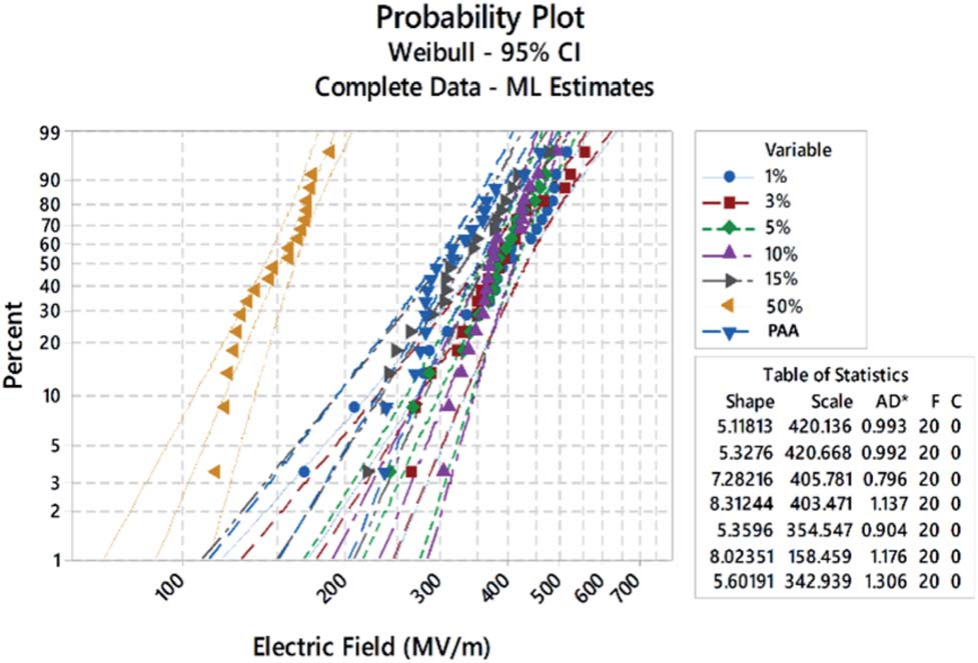
DC breakdown strengths in Weibull distribution.

**Fig. 5:**
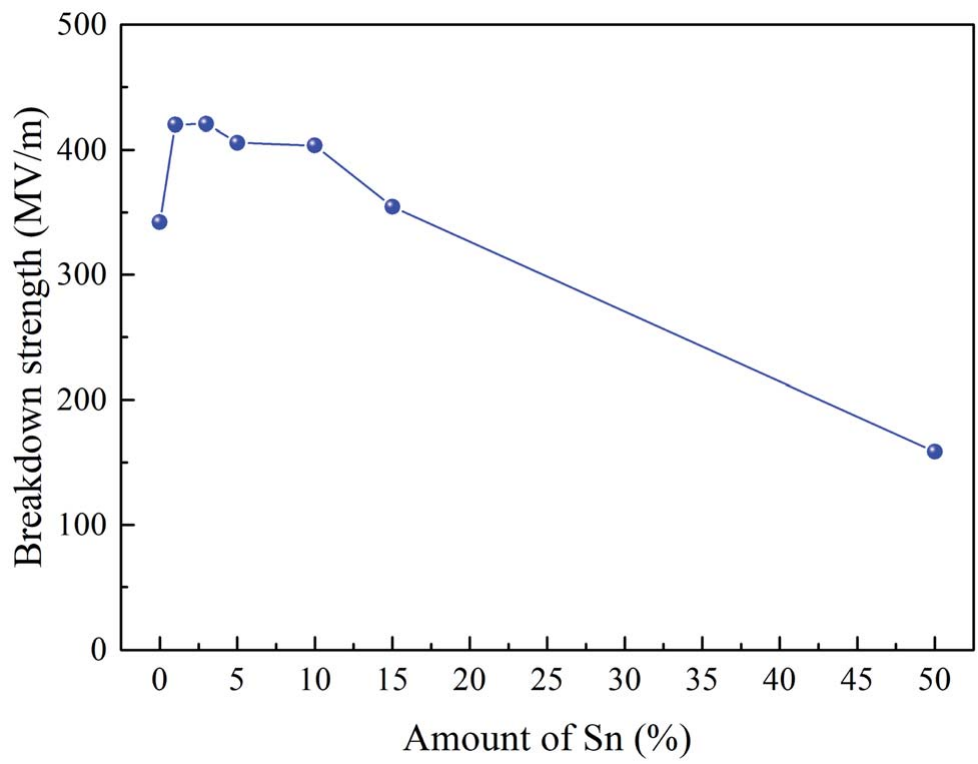
Breakdown strengths with different mol% of Sn inside the polymer.

DC breakdown strength tests were carried out on samples for each Sn-PAA system to determine the DC engineering breakdown field of all polymers. Measurements were carried out in a ball-to-plane electrode setup in silicone oil. DC breakdown voltage largely depends on the film quality, such as surface roughness, chemical defects, and physical impurities.^30,31^ Therefore, a large distribution of data points was taken using a Weibull statistical analysis to check their failure probability to assess the effect of extrinsic factors like dust, residual solvent, small molecules, and crystallization, which are responsible for lower probability breakdown, as shown in Fig. 4. A Weibull characteristic breakdown field for a given sample was determined within acceptable limits as 63.2% breakdown probability for all the polymer samples. The Weibull characteristic breakdown strength as a function of the Sn amount is illustrated in Fig. 5. The breakdown strength of PAA with 1% Sn is significantly higher than that of pure PAA. The Sn atoms acting as crosslinking points can increase the degree of polymer crosslinking, giving rise to higher breakdown strength. With Sn increasing from 1% to 10%, polymers showed breakdown strengths above 400 MV m^−1^, and no drastic change in the breakdown field was found. Interesting features have been observed that there exists a threshold limit of 10% Sn for the breakdown strength, after which the breakdown field decreases significantly, as illustrated in Fig. 5. 15% Sn containing polymers showed a breakdown field of ∼354 MV m^−1^, which is close to that of the PAA. The lowest breakdown strength was observed for the 50% Sn containing polymers. Probably because such a high amount of Sn made the film brittle, leading to the lowered breakdown strength.

The band gap between the valence band and conduction band intrinsically affects the breakdown strength of dielectric materials.^32,33^ The pure PAA film showed the lowest band gap energy. Band gap energy jumped significantly by ∼0.35 eV with the addition of 1% Sn. Up to 5% addition of Sn did not show any drastic difference in the band gap energy. Also, 10% and 15% Sn containing systems were found to have similar, and the highest overall band gap energies, respectively. The highest band gap energy measured was for the Sn10-PAA system, which inevitably had the lowest *λ*_onset_ value in nm. These results are all displayed in Table 1 below. It is relevant to note that this trend nearly matched with the trend found in the engineering DC breakdown field strength shown in Fig. 5 previously.

**Table 1:** Experimental band gap data for the Sn-PAA system along with PAA

	PAA	Sn1-PAA	Sn3-PAA	Sn5-PAA	Sn10-PAA	Sn15-PAA	Sn50-PAA
*λ* (nm)	379	344	346	339	322	325	340
*E*_g_ (eV)	3.27	3.60	3.58	3.66	3.85	3.82	3.64

## Conclusions

A new synthetic route for the creation of Sn containing polyamic acid complexes has been proposed in this work. The resultant polymers were structurally and electrically characterized for dielectric applications. The varying content of Sn starting from 1 to 50% is introduced to investigate the role of Sn as it pertains to impacting the dielectric properties of our organometallic system including dielectric constant, loss, and breakdown strength of insulating material. Comparisons of model compounds to their polymeric counterparts indicate that Sn coordinates with the carboxylate functional groups of the PAA's by bridging two strands of PAA together as Sn polyesters. This phenomenon allows for dielectric constants to increase with increasing amounts of Sn while maintaining the minimal dielectric loss. Sn helped the PAA to become more amorphous up to a threshold concentration of approximately 10%, after which the polymer began significantly increasing in crystallinity. This is evidenced by the breakdown strength, which improves upon the addition of Sn to PAA until the threshold concentration of about 10% Sn inclusion is met. This study shows that one can simultaneously improve the dielectric constant and breakdown strength of an organic PI by incorporating tiny amounts of metal into it, unlike nanocomposites which have the downfalls of aggregation and polarization loss. This system can be considered as a prototype for polymer films having a metallo–organic backbone for capacitor applications.

## Conflicts of interest

The authors declare no competing interests.

## Supplementary Material

RA-012-D1RA06302B-s001
