# Supplementary material for: High dielectric constant and high breakdown strength polyimide via tin complexation of the polyamide acid precursor
Source: RSC Adv. 2022 Mar 23;12(15):9095–100. doi: 10.1039/d1ra06302b (PMC8985109; doi:10.1039/d1ra06302b)
Supplement: RA-012-D1RA06302B-s001 [file RA-012-D1RA06302B-s001.pdf]

Electronic Supplementary Material (ESI) for RSC Advances.
This journal is © The Royal Society of Chemistry 2022

Supporting Information

High Dielectric Constant and High Breakdown Strength Polyimide via Tin Complexation of the Polyamide Acid Precursor

Abdullah Alamri [a]‡, Chao Wu [b]‡, Shamima Nasreen[c]‡, Huan Tran[d] ,Omer Yassin[c], Ryan Gentile [a], Deepak Kamal[d], Rampi Ramprasard[d], Yang Cao *[b], and Gregory Sotzing *[a c]

[a] Materials Science Program, University of Connecticut, Storrs, CT 06269, United States

[b] Electrical Insulation Research Center, Institute of Materials Science, University of Connecticut, Storrs, CT 06269, United States

[c] Chemistry Department, University of Connecticut, Storrs, CT 06269, United States

[d] School of Materials Science and Engineering, Georgia Institute of Technology, Atlanta, Georgia 30332, United States

* Corresponding authors. yang.cao@uconn.edu (Y. Cao), g.sotzing@uconn.edu (G.A. Sotzing).

‡ These authors contributed equally to this work.

Experimental

Materials

Unless otherwise specified, all chemicals and reagents were used as received without further purification. 3,3',4,4'-benzophenone tetracarboxylic dianhydride (BTDA, >98.0%), 1,6-diaminohexane (HDA, >99.0%), dimethyl tin chloride (DMT, >99.0%), and triethylamine (TEA, >99.0%) were purchased from TCI. N-methyl-2-pyrrolidone (NMP, anhydrous, 99.9%), dimethyl sulfoxide (DMSO, anhydrous, 99%), dimethylacetamide (DMAc, anhydrous, 99%), and Isopropanol were all purchased from Fisher Scientific. The BTDA was purified by sublimation under vacuum (0.03 torr). Triethylamine was purified by drying it with KOH and distilled before use.

Instrumentation

Fourier Transform Infrared (FTIR) spectra were recorded with a Nicolet Magna 560 FTIR spectrometer (resolution 0.35 cm^{-1}). Thermogravimetric analysis (TGA) was performed with TA instruments TGA Q-500 (heating rate 10 ^{o}C min^{-1} under nitrogen), and differential scanning calorimetry (DSC) analysis was performed by TA instrument DSC Q-100 with the glass transition and melting point determined from the second heating cycle with a heating rate of 10 °C min^{-1}. The diffraction data was recorded on Bruker D2 Phaser with Cu-Kα (λ=1.54184 Å) source beam with an accelerating voltage of 30 kV, and cathode current of 10 mA. The data were collected in the range of 5 to 70° 2θ values with a step size of 0.02°. Polyimide films with a thickness of ~10 microns were prepared by solution casting on borosilicate glass plates using Erichsen CoatMaster Film Applicator.

Dielectric spectroscopies were obtained using a frequency Domain Dielectric Spectrometer, Agilent 4284A Precision LCR (Inductance Capacitance Resistance) meter. Measurements were made in an air circulating oven at a constant temperature by taking frequency scans from 20 Hz to 1 MHz, with 10 volts AC voltage applied. The UV-vis spectrum for band gap determination was obtained using Varian Cary UV-VIS 5000. The UV-vis spectra were recorded from 200–800 nm and the onset wavelength of absorption, λ_{onset}, was used to determine the band gap (E_g) from Planck's equation: $E_g = hc/\lambda_{onset}$. Breakdown strength measurements were performed using a linear voltage ramp of 300 V/s

generated by a resistor capacitor (RC) circuit. The sample thickness was determined using a thickness gauge (Model LE1000-2, MeasureItAll) as the average of several measurements near the breakdown site.

Synthesis of Polymers with different Sn mole% ratios

To a dry 100 ml three-neck flask 1.162 g (10.00 mmol) of 1,6-diaminohexane (HDA), and 20 ml anhydrous NMP were added under nitrogen with stirring. After well stirred for 30 min, 3.221 g (10.00 mmol) of 3,3',4,4'-benzophenone tetracarboxylic dianhydride (BTDA) were added. The reaction was carried out at room temperature for 6 hours. Approximately 10% solution of polyamic acid (PAA) was taken for further treatment with DMT. The PAA was deprotonated with TEA before treating it with Sn. Six polymer samples were synthesized starting from 1, 3, 5, 10, 15, and 100% mole ratios of Sn as DMT with respect to PAA. For a 1 mol% Sn (DMT) containing polymer (Sn1-PAA), 0.0221 g (0.218 mmol) of TEA was added to the solution of PAA while stirring for the deprotonation of 0.2 mmol of PAA. To an Erlenmeyer flask, 0.0235 g (0.107 mmol) of $DMTCl_2$ was taken and dissolved in 10 mL of NMP. The solution was then added to the previously treated PAA solution. The reaction was carried out at room temperature for an additional 12 h. After making PAA the reaction heated up to 120 ºC for 6h. The resultant mixture was poured into 150 mL of isopropanol, filtered and washed with isopropanol several times and dried in vacuum at 110 °C for one day. The yellowish white solid was obtained as the end product. Other polymers with 3, 5, 10, 15 and 50 mol% of Sn were synthesized in a similar manner and named as, Sn1-PAA, Sn1-PAA Sn3-PAA, Sn5-PAA, Sn10-PAA, Sn15-PAA, Sn50-PAA, respectively. The 50 mol% Sn refers to the 1:1 stoichiometric ratios of Sn and PAA and hence named as Sn-PAA. Scheme.1 shows the synthetic route of Sn-PAA complex formation.

Film processing

The resultant polymer was further treated with *m*-cresol solvent to process as a drop cast film. 10 wt% solution in m-cresol was made under heated (90 ºC) condition for each

polymer. The solution was then drop casted on stainless steel metal shim stock or on a glass substrate using doctor's blade coater with a 250 µm film applicator with an application speed of 3 mm/cm. Films were dried on doctor's blade in real time environment starting from 60 ºC for one hour, and then gradual ramp of 70, 80 ºC for one hour each and finally at 90 ºC for 12 h. The films were then vacuum dried at 115 ºC for one day for the complete removal of the solvent. After one day the dried polymer films were peeled off as free-standing films. These systems would be able to produce free standing film of uniform quality, with high transparency and without brittleness up to 15% concentration of Sn content in the systems. The films were then tested for all the structural and electrical characterizations. For physical mixture samples these mixtures were processing same as the film processing mention above, then dried under vacuum at 115 ºC overnight like the polymers. 10% solutions in m-cresol were made of each mixture and casted on glass slides and dried in air for one day and under vacuum again at 115 ºC for overnight to remove the solvent for XRD analysis.

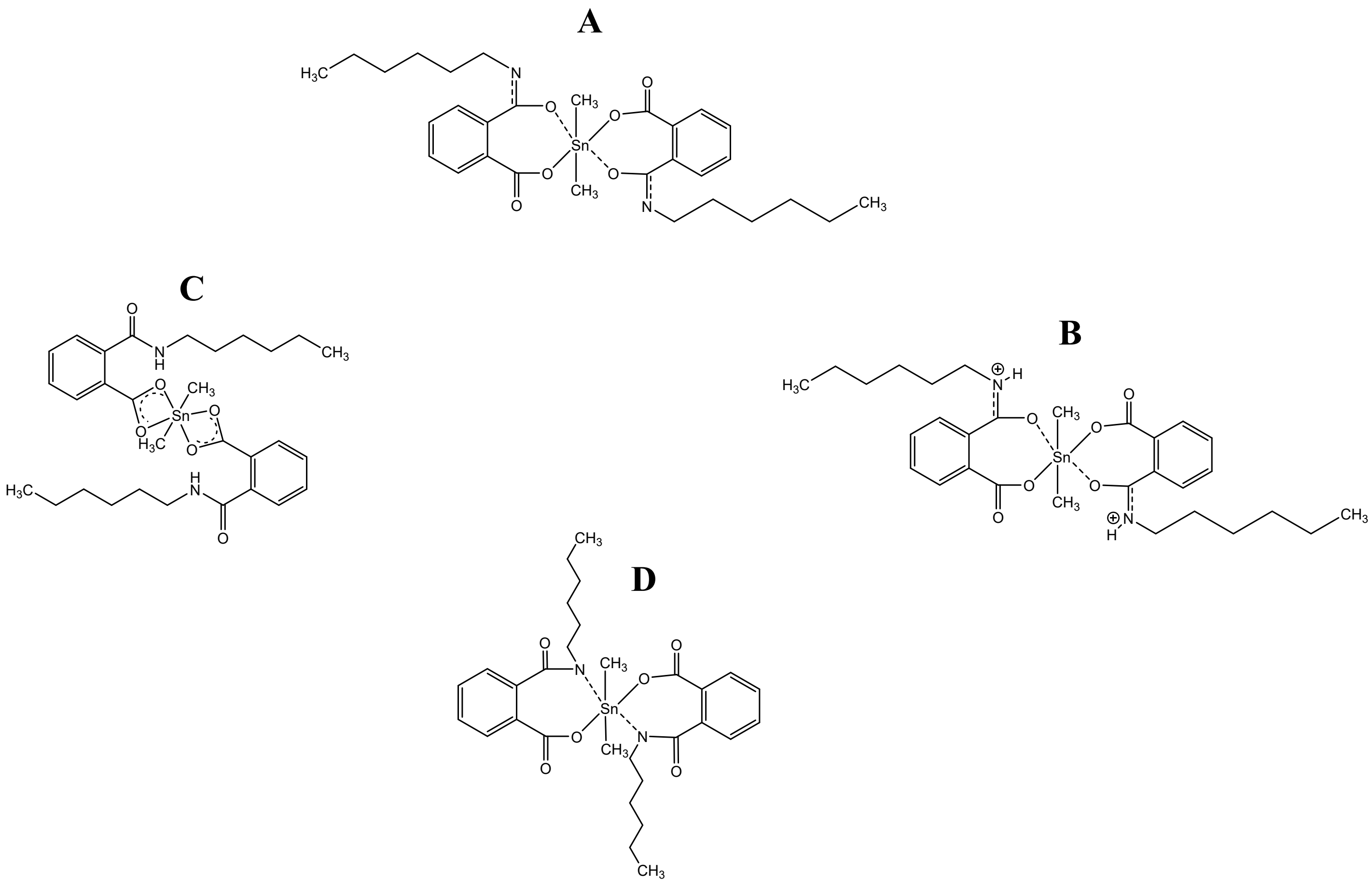

Figure 1S: Four possible complex structures predicted by DFT system.

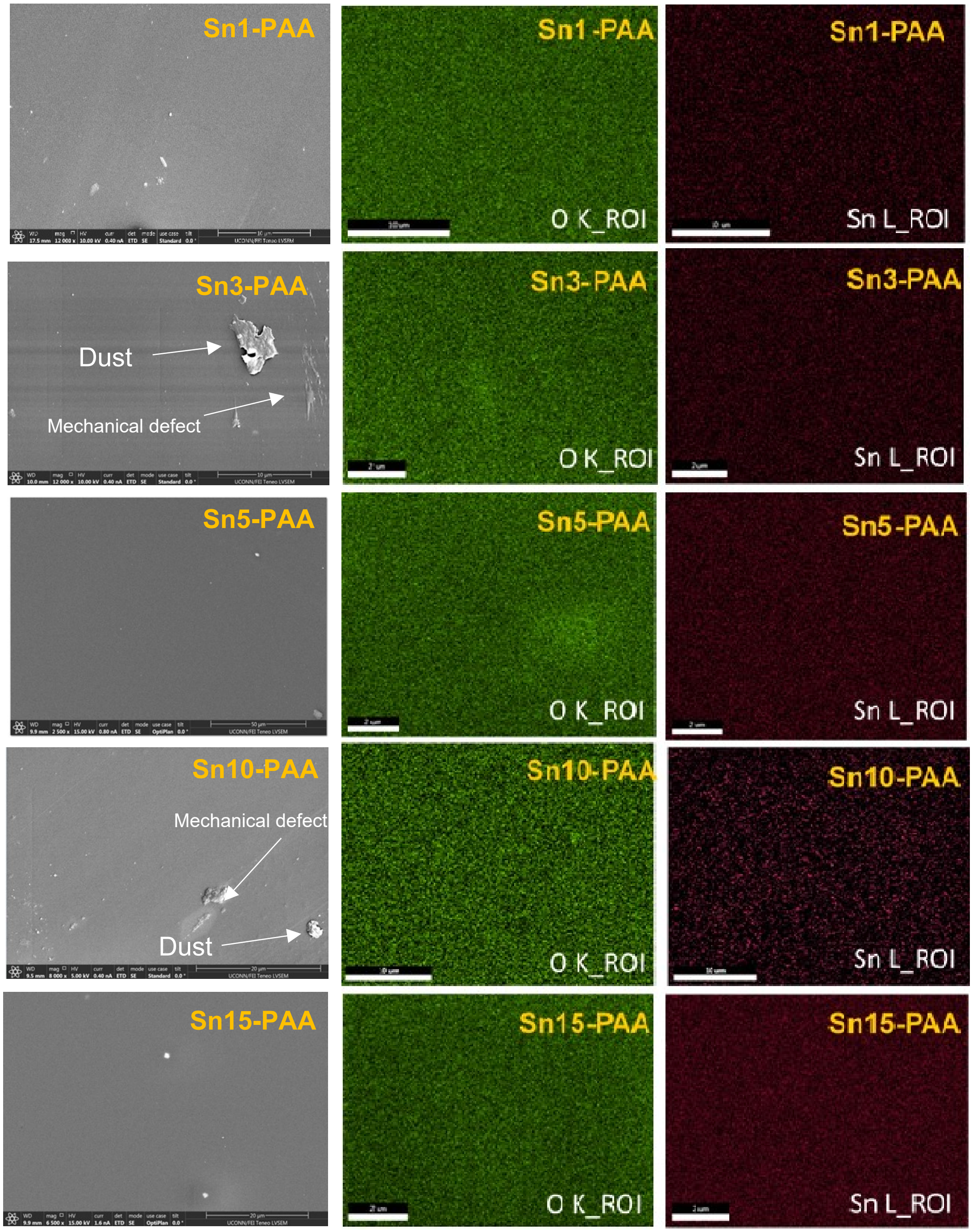

Figure 2S: (left) FE-SEM images of the films, (middle) elemental mapping by EDX showing distribution of oxygen in green dots, (right) elemental mapping by EDX showing distribution of Sn in red dots.

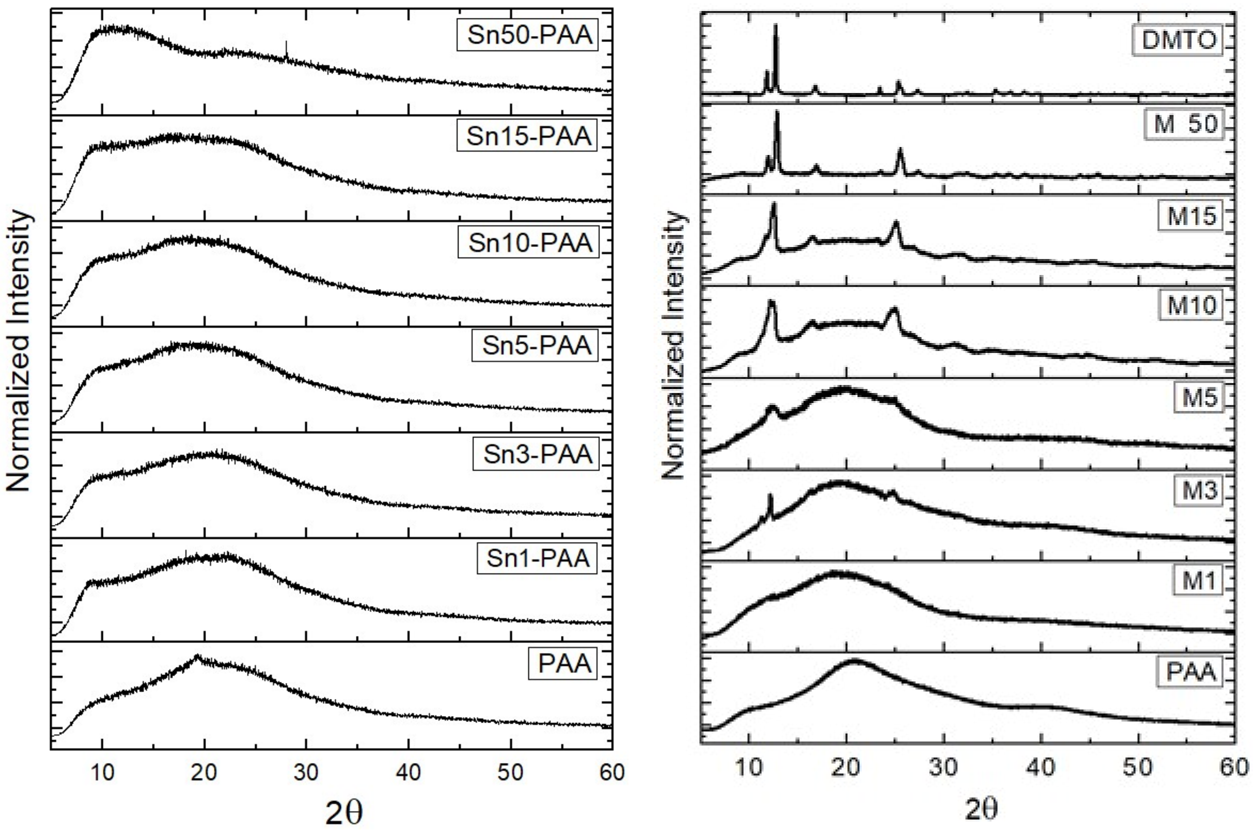

Figure 3S: XRD patterns of polymer films of Sn-PAA (left) and physical mixtures of DMTO (right) with PAA to have a comparison of structural and morphological features.

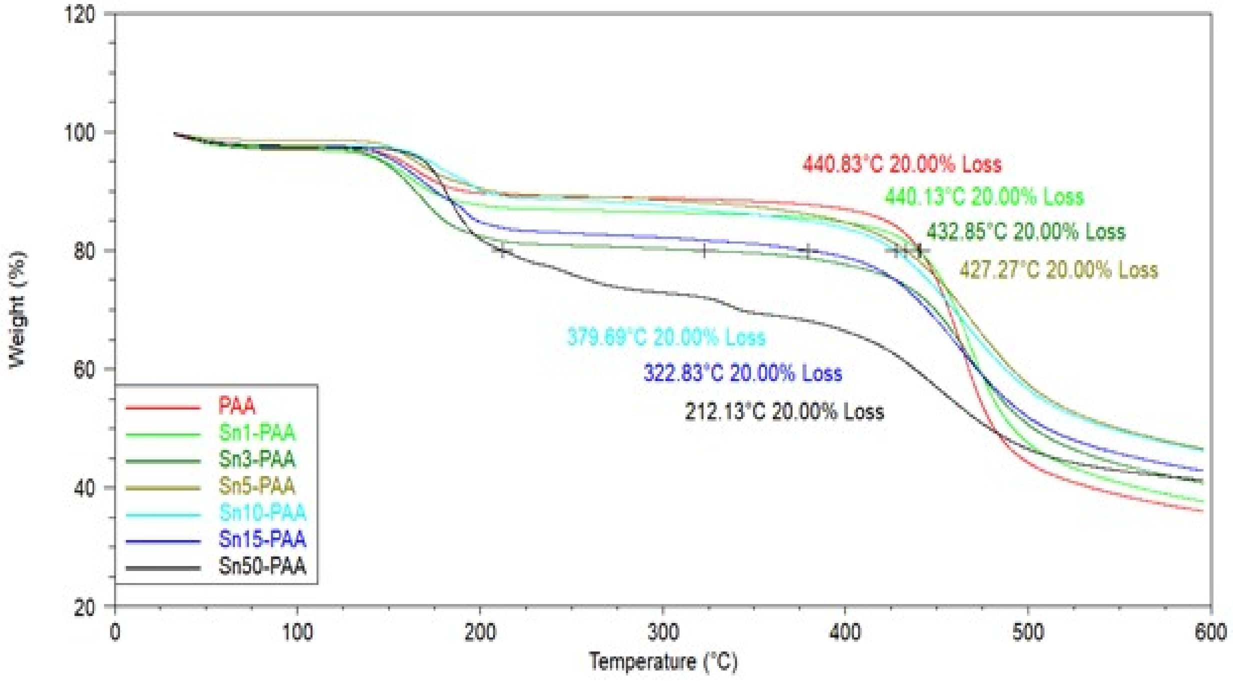

Figure 4S: Overlay curve of thermogravimetric analysis (TGA) under nitrogen atmosphere for the different Sn-Polymer weight percent.

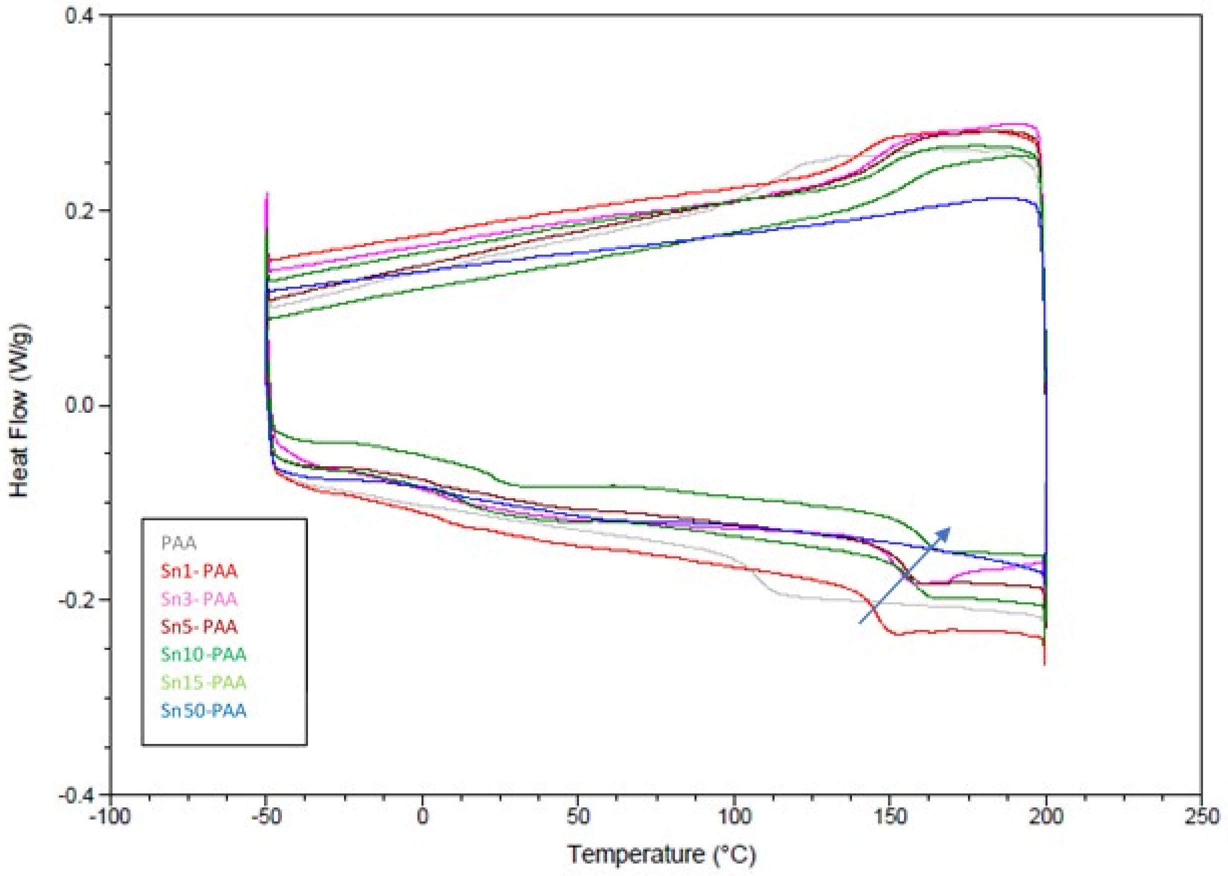

Figure 5S: Overlay DSC curves for complex polymers.

Table 1S: Thermal properties of the polymer samples

Polymer	T_g (ºC)	$T_{d20\%}$ (ºC)
PAA	107	440
Sn1- PAA	145	440
Sn3- PAA	151	432
Sn5- PAA	155	427
Sn10- PAA	157	379
Sn15- PAA	160	322
Sn50- PAA	-	212

Table 2S: Comparison between PI-Sn with other industrial polymers using for capacitors applications

Polymer	Dielectric Constant 1kHz	Breakdown (MV/m)	Band Gap Energy (eV)	Reference
BOPP	2.2	730	7	1,3
PET	3.3	570	3.8	1
PEEK	3.1	320	3.1	1
PPS	3	550	3.9	1
Polyetherimide	3.1	500	3.24	2
Polyimide	2.9	395	3.2	3,4
Tin polyester *	6.6	500	6	3,4,5
Tin polyimide 10%	5.7	404	3.85	This work

*Tin polyester is p[DMT(DMG-co-Sub)] with 80% content of DMG moity.

References:

1 G. M. Treich, M. Tefferi, S. Nasreen, A. Mannodi-Kanakkithodi, Z. Li, R. Ramprasad, G. A. Sotzing and Y. Cao, *IEEE Trans. Dielectr. Electr. Insul.*, 2017, **24**, 732–743.

2 N. Pfeiffenberger, F. Milandou, M. Niemeyer, T. Sugawara, M. Sanner and J. Mahood, *IEEE Trans. Dielectr. Electr. Insul.*, 2018, **25**, 120–126.

3 G. M. Treich, S. Nasreen, A. Mannodi Kanakkithodi, R. Ma, M. Tefferi, J. Flynn, Y. Cao, R. Ramprasad and G. A. Sotzing, *ACS Appl. Mater. Interfaces*, 2016, **8**, 21270–21277.

4 S. Nasreen, M. L. Baczkowski, G. M. Treich, M. Tefferi, C. Anastasia, R. Ramprasad, Y. Cao and G. A. Sotzing, *Macromol. Rapid Commun.*, 2019, **40**, 1–6.

5 S. Nasreen, G. M. Treich, M. L. Baczkowski, A. K. Mannodi- Kanakkithodi, A. Baldwin, S. K. Scheirey, Y. Cao, R. Ramprasad and G. A. Sotzing, *Polymer (Guildf).*, 2018, **159**, 95–105.
